# Assessment of the educational value of endodontic access cavity preparation YouTube video as a learning resource for students

**DOI:** 10.1371/journal.pone.0272765

**Published:** 2022-08-10

**Authors:** Ahmed Jamleh, Shouq Mohammed Aljohani, Faisal Fahad Alzamil, Shahad Muhammad Aljuhayyim, Modhi Nasser Alsubaei, Showq Raad Alali, Nawaf Munawir Alotaibi, Mohannad Nassar

**Affiliations:** 1 Restorative and Prosthetic Dental Sciences, College of Dentistry, King Saud bin Abdulaziz University for Health Sciences, National Guard Health Affairs, Riyadh, Saudi Arabia; 2 King Abdullah International Medical Research Centre, National Guard Health Affairs, Riyadh, Saudi Arabia; 3 College of Dentistry, King Saud bin Abdulaziz University for Health Sciences, National Guard Health Affairs, Riyadh, Saudi Arabia; 4 College of Dentistry, Prince Sattam Bin Abdulaziz University, Al-Kharj, Saudi Arabia; 5 Department of Preventive and Restorative Dentistry, College of Dental Medicine, University of Sharjah, Sharjah, United Arab Emirates; International Medical University, School of Pharmacy, MALAYSIA

## Abstract

**Objective:**

To evaluate the educational value of YouTube as a learning tool for dental students regarding endodontic access cavity preparation.

**Methods and findings:**

YouTube search was made for videos related to endodontic access cavity preparation using specific terms. After exclusions, 41 videos were chosen and assessed for tooth type, video length, days since upload, country of origin, number of views and likes, source of authorship, and viewing rate. To grade the content of videos, a usefulness score with seven elements was developed. Each element was given a score of 0 or 1. Statistical tests were run by using Kruskal-Wallis and Mann-Whitney tests (SPSS Inc, Chicago, IL, USA) at a 5% significance level. The videos received a mean of 181198.5 views with a mean duration of 686.1 seconds. The mean number of “likes” was 1047.8. Almost half of the videos covered content related to molar teeth. Most videos were provided by health care professionals with almost 50% uploaded from India. The mean usefulness score was 4.29 (range: 1–7) and the most discussed elements were description, instruments used, access cavity demonstration, and evaluation criteria. About a quarter of the videos were classified as good, while 46.3% as moderate and 29.3% as poor. Among the content usefulness categories, no difference was found in the video demographics (p*>*0.05) except “days since upload” (p = 0.018) in which good quality videos were found to have the highest median. Moreover, although insignificant, good videos were found to have the longest duration and lowest number of views, likes, and viewing rate. The mean usefulness score of videos released during the COVID-19 pandemic was lower than that for pre-pandemic videos (p = 0.042), and videos uploaded by academic institutions had a higher mean usefulness score than videos uploaded by health care professionals (p<0.001).

**Conclusions:**

Information on endodontic access cavity preparation is not comprehensive in most of the reviewed YouTube videos and could be of low educational value.

## Introduction

One of the primary objectives of root canal treatment is eradicating bacteria and managing apical periodontitis through chemo-mechanical debridement of the root canal system (RCS) [1]. To fulfill this objective, an access cavity that permits unimpeded RCS treatment is required. The American Association of Endodontists defines the access cavity as the opening prepared in a tooth to gain entrance to the RCS for 3D cleaning, shaping, and obturating. The aims of the access cavity include but are not limited to the removal of caries and pulp floor, locating the canals orifices, establishing an unconstrained path of the instruments to the canals, and preservation of tooth structure [2]. Root canal treatment consists of several phases and access cavity preparation is considered the first and very crucial technical step which if done improperly can lead to subsequent failure of the treatment [3]. Unfortunately, access cavity preparation is one of the treatment stages that students fear the most, and despite their satisfaction with the classes given on this step, it is perceived as stressful and challenging due to the complex and variant internal anatomy of teeth and the limited visualization of the pulp chamber [4, 5], hence it is quite common to encounter mishaps during this phase [6] and this might lead to the unpleasant learning process and lack of confidence in performing root canal treatment among students [7].

Preclinical endodontic education is an integral part of the overall endodontic curriculum for undergraduate dental students, through which they get exposed for the first time to the hands-on training of access cavity preparation on natural or plastic anterior and posterior teeth under the supervision of specialists or by general dental practitioners with special skills in endodontics [8, 9]. Dental educators have strived to explore new methods to accelerate the learning curve and help students learn skills faster in a preclinical setting [10]. Demonstration through face-to-face learning of the clinical procedure is the most commonly used approach [11]; however, several other methods have been described such as live-patient demonstration [12] or computer-assisted learning [10]. Nowadays, with the growth of resources, the process of gaining information has changed and the notion that certain educated people are the only source of information has been disputed [13]. Thus, in many instances, students are taking the responsibility for learning and seeking information from resources other than the bookshelves of academic libraries [14, 15]. In fact, the majority of undergraduate students excessively depend on popular internet search engines to attain further knowledge and they might steer clear of library subscription databases and electronic resources due to the complexity of library search tools [16]. The use of videos is considered an effective tool for education and has evolved into an essential part of higher education [17, 18].

YouTube, an online video platform, has been reported as the second most visited website in the world and probably the most popular video hosting website, and the large-scale acceptance of YouTube requires considerable attention from academia [19]. Indeed, dental students rely on YouTube for their learning and view it as one of the most helpful resources [20, 21]. Recently, Fu et al. [22] found that the majority of undergraduate dental students (96.7%) use YouTube as a learning tool with the main focus on learning about endodontics to improve their confidence and comprehend the steps of the procedure. This high reliance on YouTube as a tool for learning has probably been amplified due to the coronavirus pandemic.

The number of educational materials on YouTube is on a steady increase thus including it as part of education for supplementary learning material is unequivocal. However, meticulous assessment of the quality of the videos by academics is mandatory before making recommendations on its use [23]. Most of the published papers that studied the quality of endodontic-related YouTube videos analyzed the content to its usefulness as a source for patient education [24–26]. There is an obvious need for evaluating the educational value of the videos as a learning tool for dental students. Thus, this study aimed to assess the standard and usefulness of endodontic access cavity-related YouTube videos.

## Material and methods

### The strategy for YouTube search

This study was reviewed and approved by an institutional review board (NRC21R.208.05) and waived the requirement for informed consent.

On the sixteenth of December 2021, a YouTube search was run for videos related to an endodontic access cavity preparation. The following related terms were used: (1) endodontic access cavity; (2) access cavity preparation; (3) access opening; (4) outline of access cavity; and (5) shape of access cavity.

An account on YouTube was created for the purpose of the study and the included videos were stored. The search was run using an incognito window with a cache clean and unlogged browser to prevent robot learning and under default settings without any filters for sorting by relevance.

### Selection of videos

Initial screening was performed to include videos related to an endodontic access cavity preparation. Since the majority of users watch the first thirty videos [27], we stored the first thirty videos for each term. Following the duplications removal, 90 videos were then assessed. A video was excluded if one of the following criteria was observed:

- Non-English language videos
- Videos lacking written or verbal explanation
- Videos about other procedures of endodontic treatment

The collection and analysis method was complied with the terms and conditions for the source of the data.

### Evaluation of videos

The included videos were completely watched to get information about (1) tooth type, (2) video duration, (3) number of views, (4) days since upload, and (5) number of likes. The authorship source was identified as a health care professional, company, or academic institution. Also, the interactions of users were assessed based on the viewing rate by using the formula: [(number of views/number of days since upload) X 100%)].

To analyze the content of the videos in giving useful information about endodontic access cavity preparation, a scoring system of seven elements was established based on Krasner et al. [28] and Adams and Tomson [3] and consisted of the following: (1) Definition; (2) Importance of access cavity; (3) Description; (4) Instruments used; (5) Access cavity demonstration; (6) Evaluation criteria; and (7) Errors in access cavity (Table 1). Each element was scored 0 or 1 based on its consistency with the usefulness for endodontic access cavity preparation. The scoring was evaluated independently by 2 observers who were calibrated for each element. In case of disagreement, a consensus was reached after reviewing the related videos. Inter-evaluator reliability analysis was performed using the Kappa statistic to determine the variability between the evaluators.

**Table 1:** A usefulness scoring system and observation rate for videos about "endodontic access cavity preparation".

Scoring element	Score	Observation rate
Definition	1	39.0%
Importance of access cavity	1	36.6%
Description	1	92.7%
Instruments used	1	82.9%
Access cavity demonstration	1	78.0%
Evaluation criteria	1	73.2%
Errors in access cavity	1	26.8%
TOTAL	7	-

The information quality was categorized as excellent (6–7), moderate (4–5), and poor (0–3) as described by Singh et al. [29]. Statistical tests were run to compare video demographics based on the usefulness score categories, date of upload [before and after the start of the COVID-19 pandemic (date was set as March 2020)], and source of upload by using Kruskal-Wallis and Mann-Whitney tests (SPSS Inc, Chicago, IL, USA) at 5% significance level.

## Results

Of the 90 videos initially chosen, 49 were excluded as they were not in English (n = 22), lacked written or verbal explanation (n = 22), or were not related to the topic (n = 5).

The content usefulness was decided using 7 elements (Table 1). The inter-evaluator reliability for usefulness scoring was perfect (Kappa = 93.4%). The most discussed elements were “description” (92.7%), “instruments used” (82.9%), “access cavity demonstration” (78.0%) and “evaluation criteria” (73.2%), followed by “definition” (39.0%), “importance of access cavity” (36.6%) and “errors in access cavity” (26.8%) (Table 1). The mean usefulness score was 4.29 (range: 1–7) with only three videos covering the 7 usefulness elements completely.

Video characteristics are shown in Table 2. Almost half of the videos were discussing molar teeth and almost half of the videos were uploaded from India.

**Table 2:** Videos characteristics.

		n	%
**Country**	India	22	53.7
	USA	6	14.6
	UAE	5	12.3
	UK	2	4.9
	Iraq	2	4.9
	Canada	1	2.4
	Egypt	1	2.4
	Saudi Arabia	1	2.4
	Philippines	1	2.4
**Tooth Type**	Anterior	11	24.4
	Premolar	10	22.3
	Molar	24	53.3
**Source of authorship**	Health care professional	33	80.5
	Academic institution	7	17.1
	Commercial	1	2.4
**Content usefulness score**	Good (6–7)	10	24.4
	Moderate (4–5)	19	46.3
	Poor (1–3)	12	29.3

Descriptive statistics are shown in Table 3. The videos received a mean of 181198.5 views (range: 95–3028602) with a mean duration of 686.1 seconds (range: 95–3687). The mean number of “likes” was 1047.8 (Range: 0–13000).

**Table 3:** Descriptive statistics of evaluated videos (n = 41).

Demographics	Mean±SD	Median	Q1-Q3	Min-Max
Video length (in seconds)	686.1±810.4	451	203.5–727	95–3687
Days since upload	788.5±969.5	465	335–753	61–4531
Numbers of views	181198.5±492996.7	23900	5060–85886	95–3028602
Number of likes	1047.8±2153.9	271	87.5–1100	0–13000
Viewing rate	34911.1±108795.8	3819.7	1727.25–12302.2	33.5–671530.4
Content usefulness score	4.29±1.6	4	3–5.5	1–7

In terms of content usefulness score, about a quarter (24.4%) of the evaluated videos were classified as good, while 46.3% as moderate and 29.3% as poor. Among these categories, no statistically significant difference was found in video demographics (p>0.05) except “days since upload” (p = 0.018) in which good quality videos were found to have the highest median. Moreover, although insignificant, good videos were found to have the longest duration and lowest number of views, likes, and viewing rate (Table 4).

**Table 4:** Comparison of YouTube videos demographics based on the usefulness score categories.

Demographics	Poor (n = 12)		Moderate (n = 19)		Good (n = 10)		P- value^a^
	Mean±SD	Median	Mean±SD	Median	Mean±SD	Median	
Video length (in seconds)	402.01±351.7	277	640.4±691.8	420.4	1113.6±1223.0	619.5	**0.151**
Days since upload	499.5±547.6	368.5	571.2±359.6	470	1548.3±1643.2	687.5	**0.018**
Numbers of views	129330.3±203360.4	25088	264845.1±700487.5	19209	84511.9±139811.5	44212	**0.817**
Number of likes	854.3±1022.9	557	1371.1±3039.0	235	665.9±660.6	515.5	**0.602**
Viewing rate	27016.1±48838.6	6208.3	55714.1±154391.1	3711.9	4859.6±4257.8	3730	**0.282**

^a^Kruskal-Wallis and Mann-Whitney tests

The usefulness scores for videos uploaded before and after the start of the COVID-19 pandemic were 5.2±1.4 and 3.9±1.6, respectively (p = 0.042). Although insignificant, videos uploaded before the COVID-19 were found to be longer with a lower number of views, likes, and viewing rate, compared to those uploaded after the pandemic started (Table 5).

**Table 5:** Comparison of YouTube videos demographics based on the date of upload.

Demographics	Before March2020 (n = 12)		After March 2020 (n = 29)		P- value^a^
	Mean±SD	Median	Mean±SD	Median	
Video length (in seconds)	824.8±1995.9	526.7	628.7±732.7	451	**0.419**
Numbers of views	15298.7±218981.5	56922	192884.0±572613.6	21640	**0.274**
Number of likes	796.1±1061.5	292.5	1152.0±2479.2	271	**0.921**
Viewing rate	11217.4±23573.3	2729.95	44715.5±127870.4	5398.8	**0.342**
Content usefulness score	5.2±1.4	5	3.9±1.6	4	**0.042**

^a^ Mann-Whitney test

In terms of source of upload, the majority of videos (80.5%) were provided by health care professionals and 17.1% of the videos were provided by an academic institution. Only one video was uploaded by a commercial company. Videos uploaded by academic institutions showed higher content usefulness scores than those uploaded by health care professionals (p<0.001). Slightly longer videos with a higher median number of views, days since upload and likes but lower median viewing rates were observed in those provided by academic institutions (p>0.05) (Table 6).

**Table 6:** Comparison of YouTube videos demographics based on the source of upload.

Demographics	Health care professionals (n = 33)		Academic institution (n = 7)		P- value^a^
	Mean±SD	Median	Mean±SD	Median	
Video length (in seconds)	544.9±632.8	324	922.9±753.0	462	**0.069**
Days since upload	565.5±433.0	462	1649.6±1957.8	645	**0.169**
Numbers of views	201472.4±544411.6	21640	103945.6±166207.9	35492	**0.835**
Number of likes	1145.0±2376.2	271	739.4±686.2	892	**0.702**
Viewing rate	4220.6±120458.8	5139.8	5148.7±4515.1	2963.1	**0.530**
Content usefulness score	3.8±1.5	4	6±0.6	6	**0.00**

^a^ Mann-Whitney test

## Discussion

This study aimed to assess the standards and usefulness of endodontic access cavity-related YouTube videos. The overall quality of videos on access cavity preparation in terms of usefulness was found to be moderate with only few of them covering all aspects needed for comprehensive learning of the stages of the access cavity preparation.

YouTube has become an eminent video-sharing platform due to its user-friendly policy which allows free access to an unlimited number of videos without creating an account. YouTube is a favored place for students to watch and share videos, and the shift to online teaching during the COVID-19 pandemic has resulted in a further boost in its use among students [30]. Despite its potential as an instructional tool [31], the quality of YouTube videos intended for professional education has been under scrutiny [32] and this is mainly due to the lack of peer-review of the content before the online release of the videos. Video-based learning of endodontics is highly sought by students and the access cavity preparation step is the most in-demand.

Lower usefulness scores were found in videos posted after the beginning of the COVID-19 pandemic. After the declaration of COVID-19 pandemic status by the World Health Organization in March 2020, many academic institutions shifted suddenly to entirely online teaching [33]. This might have prompted fast and impromptu production of online educational materials including videos on popular social platforms such as YouTube to cope with the situation and, concurrently, students started looking for educational content online on their own without proper guidance from academics. The precise effect of this on the quality of educational videos posted during the height of the pandemic is not clearly understood. In our study, the mean usefulness score of videos posted after March 2020 was lower than earlier videos. However, interestingly enough, videos uploaded during the pandemic, though not statistically significant, had a higher number of likes, views, and viewing rates with shorter duration.

The source of the videos was classified according to its authorship as either health care professionals, companies, or academic institutions which highlights the importance and need to understand this procedure in endodontics. The mean usefulness score of videos uploaded by healthcare professionals was moderate and significantly lower than that of videos uploaded by academic institutions which was considered excellent in content. Around 80% of the evaluated videos were uploaded by health care professionals. Consistently, previous studies showed that YouTube videos for different oral-health-related topics are mostly uploaded by healthcare professionals, compared with other sources of upload [34–38]. However, there is a need for further improvement of the videos uploaded by academic institutions as only a few of them sufficiently mentioned all the needed details to produce an inclusive educational video needed for the purpose of adequate learning by the students.

In this study, more than 50% of the evaluated videos covered the access cavity preparation in molar teeth. Molar root canal treatment is considered one of the most difficult procedures for students to perform due to the complex internal anatomy of these teeth and thus several students were reported to feel insufficiently prepared to undertake root canal treatment competency assessment on molar teeth [39]. The confidence level of students in performing root canal treatment on different types of teeth was previously reported where it was the highest for anterior teeth followed by premolar and molar in descending order [40]. This might explain the focus on molar teeth in the evaluated videos of our study.

Unfortunately, errors such as perforation can occur at any stage of the access cavity preparation which might lead to low self-perceived confidence in performing the procedure [6, 7]. In the present study, for future online content, we highlight the need to adequately discuss the various types of procedural errors that might be encountered during access cavity preparation as this was missed by the majority of the evaluated videos.

Interestingly, one of the three videos that covered all elements of the usefulness score was uploaded by a dental company, and this might reflect the importance of forging collaboration between companies and academic institutions as content creators to enhance the quality of educational videos posted on YouTube and other social platforms. Furthermore, academic institutions have the responsibility to guide students to appropriate learning resources including online videos.

The results of this study highlight the importance of meticulous evaluation of the online resources by educators before recommending them to students which also may enhance the extent to which educators influence student choices on the selection of useful online resources throughout the learning process. Moreover, educators might be prompted to cooperate to convert their educational materials into videos to expand their teaching methodologies.

## Conclusion

There are a very small number of videos on YouTube with adequate information on endodontic access cavity preparation. Since the majority of videos did not show comprehensive information, dental students should not depend on YouTube as the main source of information on endodontic access cavity preparation. Academic institutions should enrich the content of YouTube with good quality videos by providing comprehensive and evidence-based information which can affect students’ knowledge and perception.

## Supporting information

S1 Table Data collection sheet for YouTube videos about endodontic access cavity preparation used as a learning resource for students. (XLSX)
